# Cobalt Phosphide (Co_2_P) with Notable Electrocatalytic Activity Designed for Sensitive and Selective Enzymeless Bioanalysis of Hydrogen Peroxide

**DOI:** 10.1186/s11671-020-03469-9

**Published:** 2021-01-13

**Authors:** Donghang Yin, Junyan Tang, Rongbiao Bai, Shuyi Yin, Mengnan Jiang, Zigui Kan, Hongmei Li, Fei Wang, Caolong Li

**Affiliations:** 1 Key Laboratory of Biomedical Functional Materials, School of Science, China Pharmaceutical University, Nanjing, 211198 People’s Republic of China; 2 Tibetan Medicine Research Institute, Tibetan Traditional Medical College, Lhasa, 850000 Tibet People’s Republic of China

**Keywords:** Cobalt phosphide, Hydrogen peroxide, Non-enzymatic, Amperometric sensor

## Abstract

In this work, cobalt phosphide nanoparticles (Co_2_P NPs) were prepared by simple and mild hydrothermal method without the use of harmful phosphorous source. The morphological structure and surface component of Co_2_P were characterized by transmission electron microscopy, X-ray diffraction and X-ray photoelectron spectroscopy measurements. Considering the excellent electrocatalytic reduction activity and good electrical conductivity of transition-metal phosphide, we fabricated Co_2_P NPs on indium tin oxide (ITO) substrate (Co_2_P/ITO) for H_2_O_2_ detection. The Co_2_P/ITO transducer displayed a rapid amperometric response less than 5 s, a broader response range from 0.001 to 10.0 mM and a low detection limit of 0.65 μM. In addition, the non-enzymatic Co_2_P/ITO sensor showed outstanding selectivity, reproducibility, repeatability and stability, all of which qualified the Co_2_P/ITO electrode for quite a reliable and promising biosensor for H_2_O_2_ sensing.

## Introduction

Hydrogen peroxide (H_2_O_2_) is a representative reactive oxygen species in living organisms, and it plays a critical role in normal physiologic function [1]. The concentration of H_2_O_2_ in living cells is related closely with the cell physiological balance [2]. Numerous studies have also been reported that cancer, Alzheimer’s diseases, Parkinson’s diseases and some severe diseases may be caused by abnormal concentration of H_2_O_2_ [3–5]. Developing accurate, sensitive, rapid and selective methods to detect the concentration of H_2_O_2_, a normal oxidative stress biomarker, will be undoubtedly beneficial to the early diagnosis. Up to now, a host of analytical methods such as spectroscopy [6], colorimetry [7], fluorescence [8, 9] and electrochemical methods [10–12] have been applied in H_2_O_2_ determination. Electrochemical method, especially amperometric test is gradually becoming one of the most simple and effective detection methods for H_2_O_2_ biological analysis among diverse sensing methods due to its advantages such as high sensitivity, outstanding selectivity and low cost.

Enzymatic electrochemical sensors have been proved to be effective instruments for detecting H_2_O_2_. However, the large-scale practical application of enzyme-based sensors is limited by complicated immobilization, environmental instability and low reproducibility. Therefore, developing non-enzymatic electrochemical H_2_O_2_ sensors is highly indispensable.

In recent years, a growing number of sensors based on noble metal [13–15], non-noble metal and their corresponding compounds [16–19] or carbon materials [20, 21] have been used for electrochemical H_2_O_2_ detection. As electrochemical active materials for fabricating non-enzymatic biosensors, transition metal compounds have been received increasing interests. Transition-metal phosphides (TMPs) are a class of newly developed materials with excellent electrocatalytic activity, good electrical conductivity and a plenty of outstanding properties. Thus, they have been extensively studied for applications in water splitting [22, 23], hydrodesulfurization [24], and supercapacitor electrodes [25]. Recent research indicates that CoP, Ni_2_P and Cu_3_P [26–28] can also be used as efficient electrocatalyst for non-enzymatic H_2_O_2_ detection. However, the number of researches about the application of TMPs in bioanalysis is still limited nowadays. Besides, the use of triphenylphosphine [29, 30], white phosphorous [31, 32] or other environmental hazardous phosphorous source [33] can increase the operational risk in the preparation of TMPs. Therefore, some research work for developing green method in TMP preparation is worth being supplemented in this area.

In this work, cobalt phosphide nanoparticles (Co_2_P NPs) were prepared by one-step hydrothermal method utilizing cobalt acetate and red phosphorous as raw materials. Herein, we fabricated Co_2_P NPs on indium tin oxide (ITO) substrate by drop-casting method for H_2_O_2_ detection. Co_2_P displayed excellent electrocatalytic activity toward H_2_O_2_ reduction. Moreover, it revealed favorable selectivity, excellent reproducibility and good stability, which therefore exhibited its potential application as a sensitive platform for H_2_O_2_ detection.

## Experimental Section

### Reagents and Materials

All reagents were analytical grade and used without further purification. Cobalt (II) acetate tetrahydrate (Co(Ac)_2_·4H_2_O), cobalt chloride hexahydrate (CoCl_2_·6H_2_O), D-(+)-glucose, L-Glycine (L-Gly), ascorbic acid (AA), uric acid (UA), urea, NaCl, KCl, NaH_2_PO_4_, Na_2_HPO_4_, hydrogen peroxide (30% H_2_O_2_), ethanol and acetone were purchased from Sinopharm Chemical Reagent Co., Ltd. China. D-(–)-fructose, L-arginine (L-Arg), L-lysine (L-Lys), dopamine (DA), acetaminophen (APAP), amino trimethylene phosphonic acid (ATMP, 50 wt%) were purchased from Aladdin Ltd. Commercial red phosphorous (98.5%, 100 mesh) were purchased from Energy Chemical Technology (Shanghai) Co., Ltd. Nafion PFSA polymer dispersion (5%) were purchased from Beijing Honghaitian technology Co., Ltd. Deionized water was used in all the experiments. The indium tin oxide (ITO) glass (10 × 20 × 1.1 mm with an ITO film of 185 ± 2 nm and a sheet resistance of 6.6 ± 0.1 Ω) was supplied from Shenzhen South Xiangcheng Technology Co., Ltd.

### Synthesis of Co_2_P Nanoparticles

Commercial red phosphorous (2 g) was dispersed in 15 mL H_2_O under sonification and hydrothermally treated at 200 °C for 12 h in a 50 mL Teflon-lined stainless autoclave to clear oxide layers [34]. Then, the hydrothermal treated red phosphorous was dried in a vacuum oven. After finishing the pretreatment of red phosphorous, 1 mmol Co(Ac)_2_·4H_2_O was dissolved in 30 mL distilled water to obtain an aqueous solution. Then, the hydrothermal treated red phosphorous was added into the solution under ultrasonication for 15 min with the molar ratio of Co/P 1/10. The prepared suspension was rapidly poured into a 50 mL Teflon-lined autoclave. Then, the autoclave was placed in an electronic oven and hydrothermally treated at 160, 200, 240 °C for 12 h, respectively. Then, the product was collected by centrifugation and washed three times with distilled water and ethanol, respectively. Finally, Co_2_P NPs were dried at 60 °C for 3 h in air.

### Synthesis of Co(PO_3_)_2_

The preparation method of Co(PO_3_)_2_ was referred to the previous report [35]. 0.1 M CoCl_2_·6H_2_O methanol solution was prepared firstly. Then, 2 mL ATMP (50 *w*t%) was added dropwise into 20 mL the above purple solution and stirred for 30 min. The insoluble cobalt-metaphosphate coordination polymer formed in the solution subsequently. The obtained pink powder was further heated to 900 °C under Ar flow with a heating rate of 5 °C·min^−1^ and then held for 2 h. After cooling down to room temperature, the black product was collected and reheated at 650 °C for 4 h in air to remove the carbonized organic ligand. Finally, the light-purple powder of Co(PO_3_)_2_ was obtained.

### Fabrication of Co_2_P/ITO Electrode

**Scheme 1.**
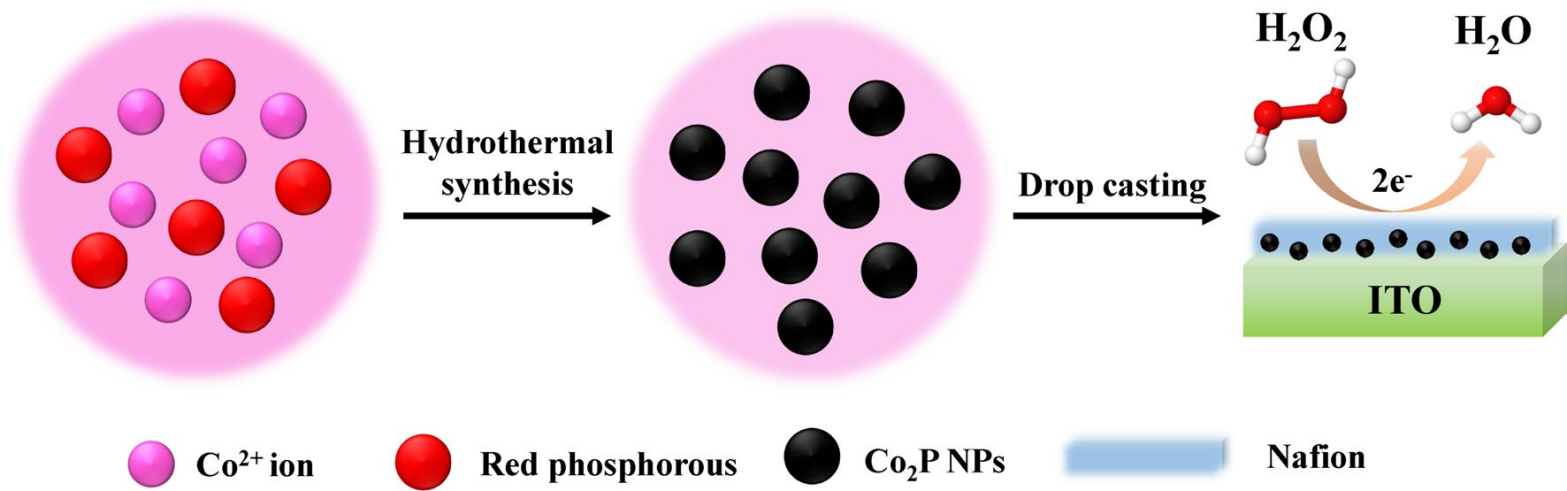
Schematic preparation of Co_2_P/ITO electrode and H_2_O_2_ sensing

Firstly, the ITO glass (1 cm × 2 cm) was cleaned in acetone, ethanol and deionized water for 10 min, respectively, by sonication. After that, the treated ITO was dried under nitrogen sweeping. For the modification of the electrode, 5 mg of the Co_2_P NPs was dispersed in 1 mL deionized water to form 5 mg mL^−1^ Co_2_P suspension. Then, 5 μL 5% Nafion solution was added into the suspension and the mixture was ultrasonicated for 15 min to obtain uniform ink-like suspension. The Co_2_P/ITO electrode was prepared by drop-casting 100 μL of Co_2_P suspension on the ITO surface, and dried in air as working electrode. The schematic preparation process of Co_2_P/ITO electrode is shown in Scheme 1.

### Characterizations

The X-ray diffraction (XRD) data were analyzed by a D8 ADVANCE diffractometer with Cu Kα radiation. The transmission electron microscopy (TEM) measurement was conducted using a Tecnai G2 F20 with energy dispersed spectrum detector. X-ray photoelectron spectroscopy (XPS) spectra were measured on a Thermo ESCALAB 250XI spectrometer.

### Electrochemical Measurements

Voltammetry measurements were accomplished by CHI 660E electrochemical workstation in a three-electrode system, employing Co_2_P/ITO electrode as working electrode, a platinum foil (1 cm × 1 cm) as counter electrode and Ag/AgCl with 3 M KCl solution as reference electrode to study the electrochemical activities of the synthesized samples for H_2_O_2_ detection. Phosphate buffer saline (PBS; 0.1 M, pH 7.4) was used as the electrolyte to simulate the physiological medium in human body. The sensing performances of Co_2_P/ITO electrode toward H_2_O_2_ detection were investigated by cyclic voltammetry (CV) and amperometry (I-t). All the detection experiments were performed under 100 rpm stirring at room temperature. Electrochemical impedance tests were performed on VersaSTAT 3F electrochemical workstation and ferricyanide solution was used as the electrolyte for impedance measurement.

## Results and Discussion

### Characterization of Co_2_P NPs

**Fig. 1:**
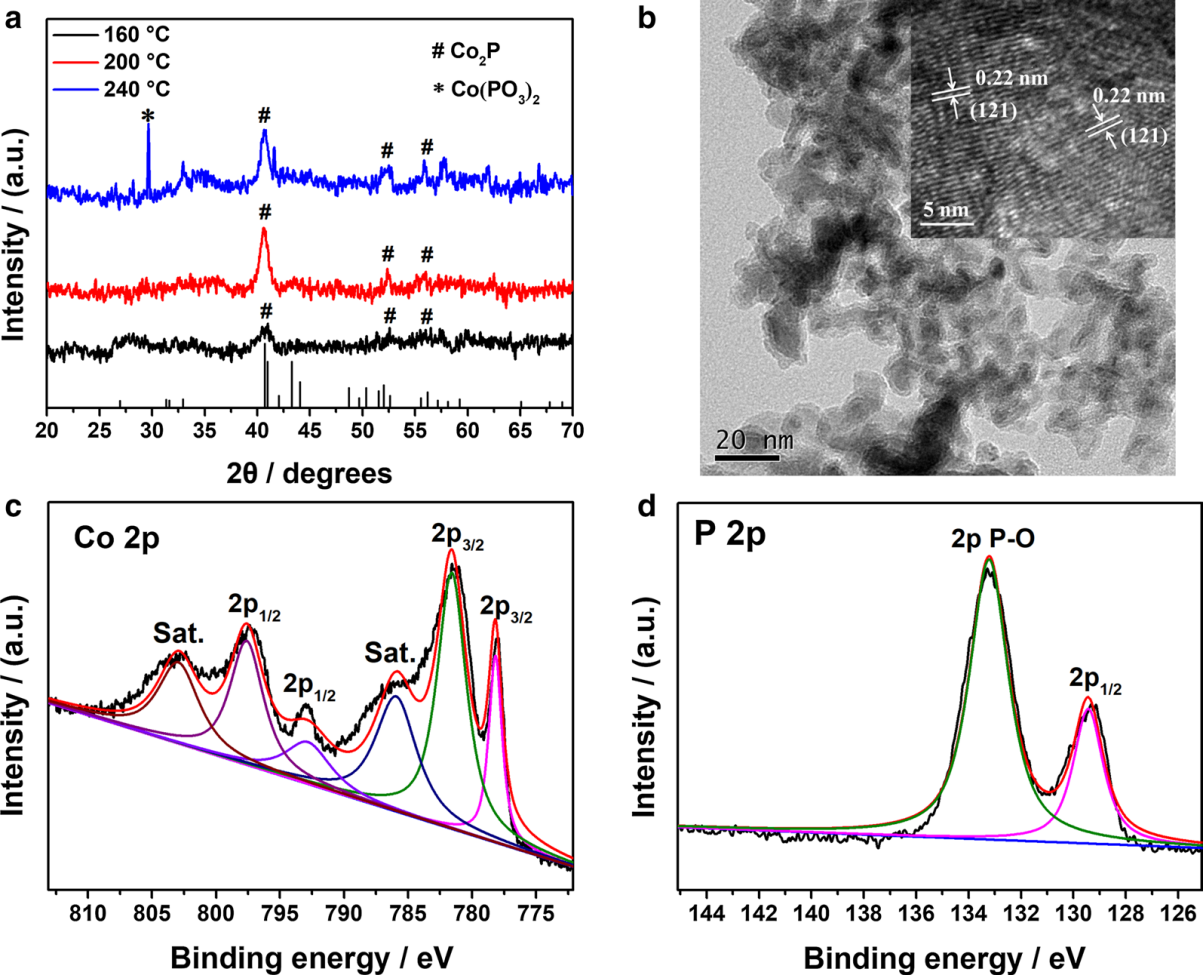
**a** XRD patterns of Co_2_P NPs prepared at different temperatures for 12 h. **b** Transmission electron microscopic image and high-resolution transmission electron microscopic image (inset) of Co_2_P NPs. XPS spectra of Co_2_P in the **c** Co 2*p* region and **d** P 2*p* region

The crystal structure of Co_2_P NPs was confirmed by XRD measurement. Figure 1a shows the XRD patterns of Co_2_P samples prepared at 160, 200 and 240 °C for 12 h. The Co_2_P sample prepared at 200 °C shows diffraction peaks at around 40.7°, 40.9°, 52.0° and 56.2° which correspond to the characteristic diffraction planes at (121), (201), (002) and (320) for the orthorhombic phase of Co_2_P (JCPDS no. 32-0306). When temperature varied from 160 to 200 °C, the intensities of diffraction peaks increased and the peaks became narrower and sharper, indicating that the products had a higher crystallinity at 200 °C. However, when temperature reached 240 °C, some impurities were formed and the diffraction peaks at 29.7° was attributed to the diffraction plane at (-222) of Co(PO_3_)_2_ (JCPDS no. 27-1120). The influence of synthetic time on the preparation of Co_2_P under 200 °C is shown in Additional file 1: Fig. S1. When the time duration was controlled within 12 h, the obtained Co_2_P NPs displayed the lowest value of full width at half maximum of (121) peak, suggesting better crystallinity. Besides, none of impurities existed in the sample when the reaction time varied from 6 to 24 h. According to the Scherrer formula, the calculated grain size of Co_2_P NPs prepared at 200 °C for 12 h was 14.2 nm.

The morphology of Co_2_P NPs was assessed by TEM measurements. As shown in Fig. 1b, the product prepared at 200 °C is composed of irregular nanoparticles with the diameter around 10–20 nm and two lattice fringes can be clearly seen in the high-resolution TEM (HRTEM) image (inset in Fig. 1b). The distance between the neighboring planes is 0.22 nm, corresponding to the (121) facets of Co_2_P, which further confirms that the formation of TMP is Co_2_P.

The XPS technique was employed in analyzing the chemical compositions on the surface of the Co_2_P. Additional file 1: Fig. S2 shows the XPS survey spectrum of Co_2_P. Co, P and O elements are detected in the sample, confirming the existence of Co_2_P and some oxidized products. Energy-dispersive X-ray spectroscopy (EDX) spectra of Co_2_P (Additional file 1: Fig. S3) further confirms the co-existence of three elements (Co, P, O) in the sample. The high-resolution XPS spectra of Co 2p and P 2p are shown in Fig. 1c, d, respectively. In Co 2p spectrum, the peaks at 781.1 and 797.6 eV can be ascribed to the binding energies (BEs) of Co^2+^ 2*p*_3/2_ and Co^2+^ 2*p*_1/2_, respectively [26, 36]. The peaks at 786.0 and 803.1 eV are two apparent shake-up satellite peaks. The Co 2p BE of 778.2 eV shifts positively from that of metallic Co (777.9 eV), which suggests that Co in Co_2_P has a partial positive charge (*δ*^+^) with a small value (0 < *δ* < 2) [37]. On the contrary, the P 2*p* BE of 129.4 eV shifts negatively from that of elemental P (130.2 eV) so that the P has a partial negative charge (*δ*^−^) in Co_2_P. The changes of BE in Co and P element compared with their elementary substance, respectively, reveal that the transfer direction of electron density in Co_2_P is from Co to P [38]. Superficial oxidation of Co_2_P generates a few of oxidized P species in the sample. Therefore, the peaks at 133.2 eV in high BE range are assigned to the oxides [39].

### Electrochemical Detection of H_2_O_2_ at Co_2_P/ITO Electrode

**Fig. 2:**
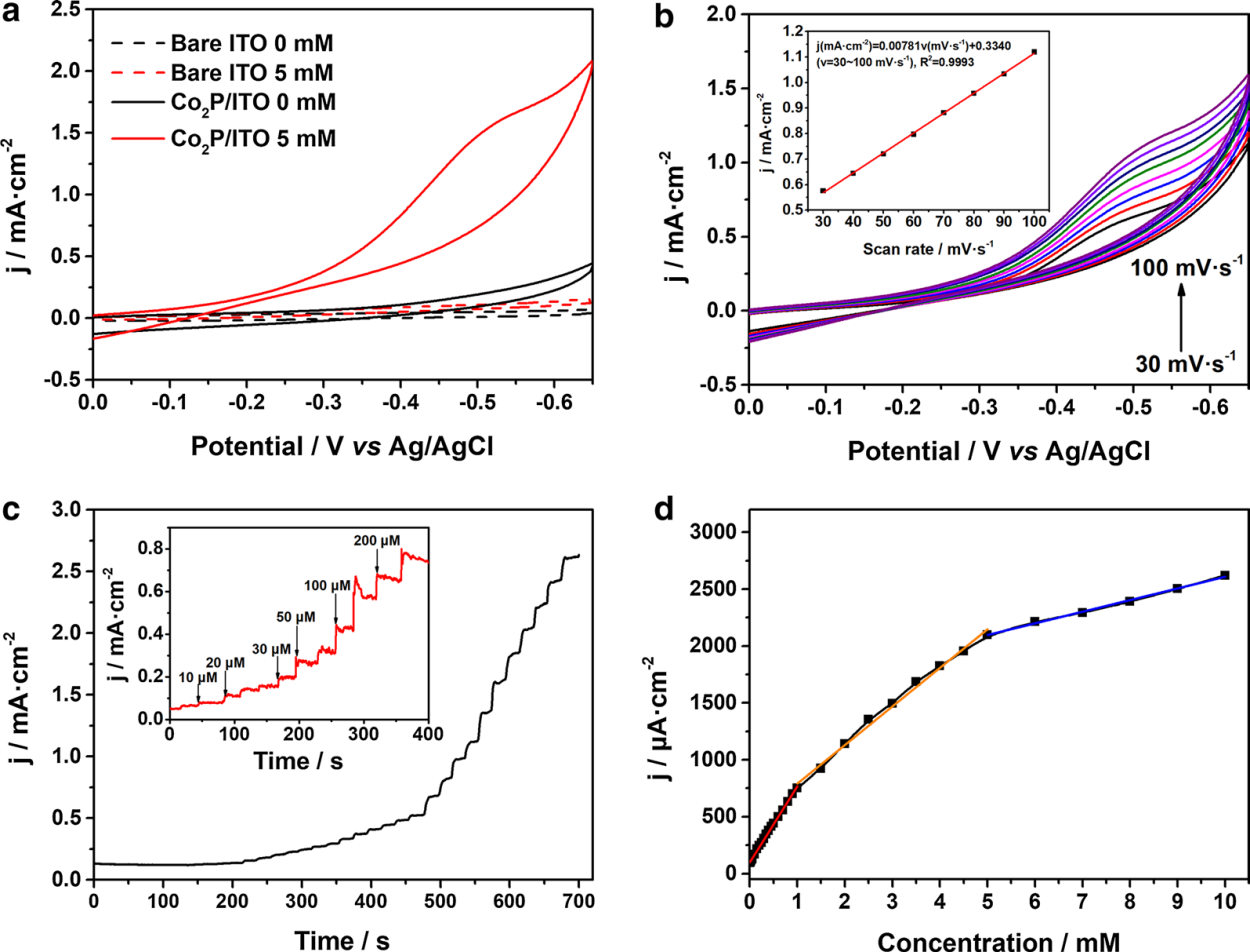
**a** CV curves of bare ITO and Co_2_P/ITO electrode in 0.1 M PBS with and without 5.0 mM H_2_O_2_ at a scan rate of 100 mV s^−1^. **b** CV curves of Co_2_P/ITO electrode in 2.5 mM H_2_O_2_ at scan rates from 30 to 100 mV s^−1^. Inset: The corresponding plot of current versus the scan rate. **c** Amperometric responses of Co_2_P/ITO electrode with successive addition of H_2_O_2_ in 0.1 M PBS. **d** The calibration curve of steady current versus the concentration of H_2_O_2_

To investigate the electrocatalytic activity of Co_2_P NPs in H_2_O_2_ reduction, we designed a non-enzymatic H_2_O_2_ electrode by drop-casting Co_2_P NPs suspension on a bare ITO surface. Figure 2a shows the CV curves of bare ITO and Co_2_P/ITO in 0.1 M PBS at pH 7.4 with and without 5.0 mM H_2_O_2_, respectively. The dash lines indicate that the response of bare ITO to H_2_O_2_ reduction is negligible. However, the Co_2_P/ITO electrode exhibits a remarkable reduction peak at − 0.5 V in the presence of H_2_O_2_, which demonstrates the prominent electrocatalytic activity of Co_2_P NPs toward H_2_O_2_ reduction. Figure 2b presents the CV curves of Co_2_P/ITO at different scan rates (from 30 to 100 mV s^−1^) with 2.5 mM H_2_O_2_. When increasing the scan rate, the reduction peak current increased and the peak potential shifted to the more negative potential side, indicating the reduction in H_2_O_2_ on Co_2_P/ITO was an irreversible reaction. The corresponding calibration curve (inset, Fig. 2b) shows that the reduction peak current densities increase linearly proportional to the scan rate, suggesting that the electrochemical reduction of H_2_O_2_ on the surface of Co_2_P/ITO electrode is a surface-controlled process [40].

Figure 2c, d show the amperometric response and the calibration curve of Co_2_P/ITO electrode upon the successive addition of H_2_O_2_ into the 0.1 M PBS at − 0.5 V with stirring. The Co_2_P/ITO electrode exhibited quick response to the addition of H_2_O_2_ and achieved the steady-state current within 5 s. The calibration curve in Fig. 2d shows that the transducer displays a multi-linear range of H_2_O_2_ concentration from 0.001 to 1.0 mM, 1.0–5.0 mM and 5.0–10.0 mM. The sensitivity of the sensor alters with the increasing concentration of H_2_O_2_, due to the change of electrocatalytic reduction kinetics of H_2_O_2_ on the electrode surface. According to previous reports, the rate-determining step of H_2_O_2_ reduction is dominated by H_2_O_2_ adsorption at low concentration, whereas the activation of H_2_O_2_ is the major determinant at high concentration. In the middle region, the reduction kinetics of H_2_O_2_ is controlled by adsorption and activation at the same time [10]. A multitude of analysts will adsorb on the surface of Co_2_P and cover the active sites in the high concentration, which lead to the decrease in sensitivity [41].

The comparison on H_2_O_2_ sensing performances of the prepared Co_2_P sample at various reaction temperature and time is shown in Additional file 1: Fig. S4, S5 and Table S1, indicating that the Co_2_P sample prepared at 200 °C for 12 h displays the best H_2_O_2_ sensing performances. When reaction temperature raised to 240 °C, the formed Co(PO_3_)_2_ in Co_2_P could be regarded as impurity. To further clarify the influence of Co(PO_3_)_2_ on H_2_O_2_ detection, the electrochemical properties of Co(PO_3_)_2_ were investigated. As shown in Additional file 1: Fig. S6, Co(PO_3_)_2_ displays negligible electrochemical response toward H_2_O_2_ and its conductivity is inferior to Co_2_P, which declines the current signal of Co_2_P/ITO in amperometric test. Therefore, the higher purity and better crystallinity of Co_2_P sample may contribute to the improvement of sensing performances. Thus, we choose the Co_2_P sample prepared at 200 °C and 12 h as the best H_2_O_2_ sensing material. The calibration *I*–*t* curve also presents a good linear relationship in the concentration of 1.0–50 μM, the physiological range of H_2_O_2_ concentration in biosystem (Fig. S7) [28], which could be helpful to improve the possibility of practical applications of this sensor. In addition, the limit of detection (LOD) of the H_2_O_2_ sensor can be calculated to be 0.65 μM at a signal-to-noise ratio of 3. Compared with the previously reported H_2_O_2_ sensor, the comprehensive electrochemical performances of our Co_2_P/ITO transducer are superior to those with favorable sensitivity, linear range and LOD, as shown in Table 1.

**Table 1:** Sensing performances on comparison of Co_2_P/ITO with other cobalt-based non-enzymatic H_2_O_2_ sensors

Materials	Linear range	Sensitivity (μA mM^−1^ cm^−2^)	Detection limit (μM)	References
Co_3_O_4_–rGO	15–675 μM	1140	2.4	[42]
Co_3_O_4_ nanowire/N-carbon foam	0.01–1.4 mM	230	1.4	[16]
Co_3_O_4_/MWCNTs	0.02–0.43 mM	1000	2.46	[43]
Co_3_O_4_/rGO	1–18.5 mM	–	0.5	[44]
CoS	0.005–14.82 mM	459	1.5	[45]
CoP NWs	0.001–12 mM	–	0.48	[26]
Hb/CoP-CC (carbon cloth)	2.0–2670 μM	56.2	0.67	[40]
Co_2_P/ITO	0.0001–1.0 mM	668.6	0.65	This work
	1.0–5.0 mM	339.0		
	5.0–10.0 mM	102.3		

**Fig. 3:**
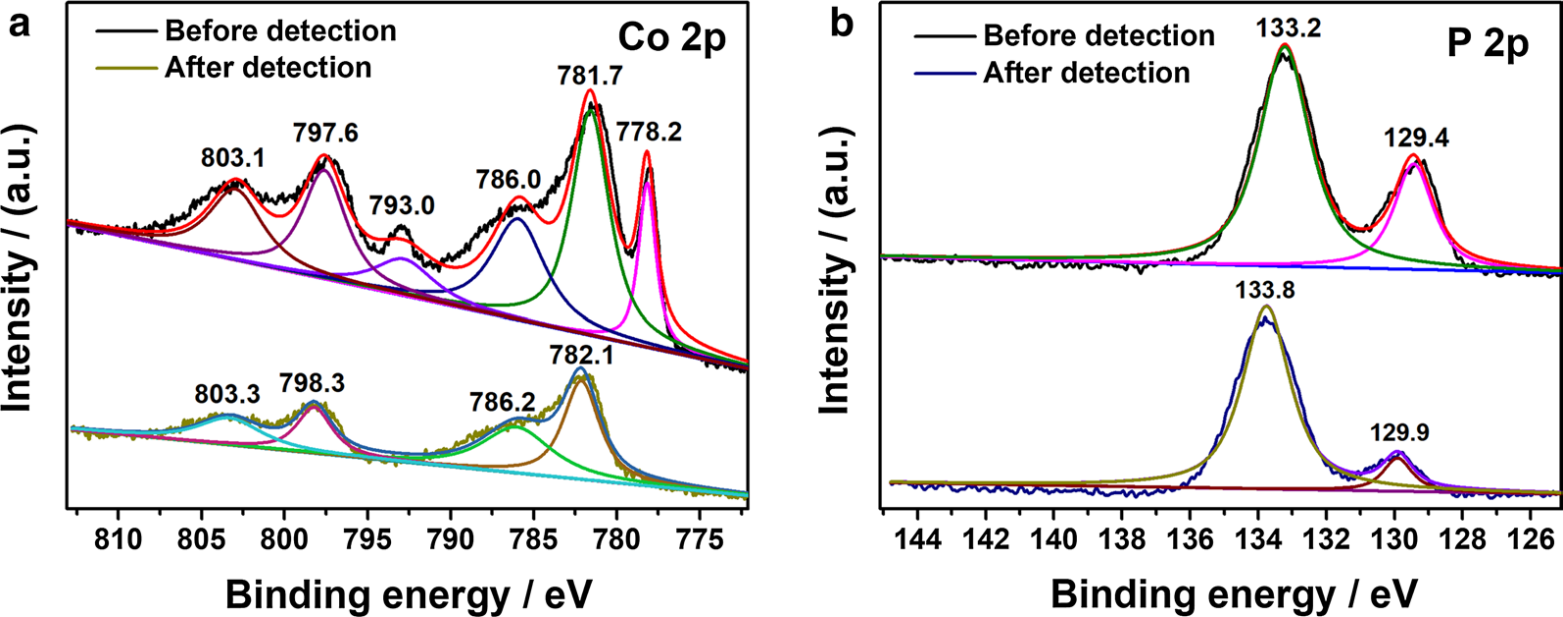
The comparison of XPS spectra in **a** Co 2*p* region and **b** P 2*p* region of Co_2_P before and after detection

After detecting 1.0 mM H_2_O_2_ repeatedly for 35 times (Fig. 3a, b), the XPS spectra in Co 2*p* and P 2*p* region of Co_2_P are analyzed to further investigate the sensing mechanism. There is no significant change in the position of the peaks in P 2*p* region before and after H_2_O_2_ detection. However, the peaks at 778.2 and 793.0 eV in Co 2*p* spectrum disappeared after multiple measurements. As the peak at 778.2 eV indicates the existence of reduced Co species in Co_2_P sample [37], the disappearance of these two peaks demonstrates that the reduced Co species with low valence in Co_2_P may be oxidized by H_2_O_2_ during the detection process, especially with high concentration of H_2_O_2_. The remnant peaks in Co 2*p* region (782.1 and 798.3 eV) are attributed to Co^2+^ 2*p*_3/2_ and Co^2+^ 2*p*_1/2_, respectively, suggesting the exclusive existence of Co(II) species in Co_2_P after multiple measurements. According to previous reports about the utilization of cobalt-based electrocatalyst in H_2_O_2_ detection, Co^2+^ species are demonstrated as the catalytic active sites for H_2_O_2_ reduction [46–48]. Generally, the electrochemical reduction in H_2_O_2_ goes through two steps in PBS [49, 50], as shown below.

1 $${\text{H}}_{{2}} {\text{O}}_{{2}} + {\text{ e}}^{ - } \to {\text{OH}}_{{{\text{ad}}}} + {\text{ OH}}^{ - }$$

2 $${\text{OH}}_{{{\text{ad}}}} + {\text{ e}}^{ - } \to {\text{OH}}^{ - }$$

3 $${\text{2OH}}^{ - } + {\text{ 2H}}^{ + } \to {\text{2H}}_{{2}} {\text{O}}$$

In the first step, H_2_O_2_ obtains an electron to form adsorbed OH^−^ (OH_ad_). When the intermediate OH_ad_ obtains an additional electron, the final reduction product of H_2_O_2_, H_2_O, is generated. As the redox potential of H_2_O_2_/H_2_O is higher than Co^3+^/Co^2+^, the Co(II) species in Co_2_P can be oxidized to Co(III) in the electron transfer process and H_2_O_2_ is reduced to H_2_O irreversibly. During the amperometric test, the applied bias is − 0.5 V versus Ag/AgCl (equals to 0.14 V vs*.* NHE), which is lower than the standard redox potential of Co^3+^/Co^2+^. As a result, the oxidized Co(III) can be reduced to Co(II) and theses catalytic active sites of Co(II) are regenerated again. Therefore, it can be concluded that the catalytic cycle of Co (II) species takes place during electrochemical detection of H_2_O_2_ and the reduced Co species with low valence are oxidized by H_2_O_2_ after repetitive measurements.

### Selectivity, Stability, Reproducibility and Repeatability of Co_2_P/ITO Electrode

**Fig. 4:**
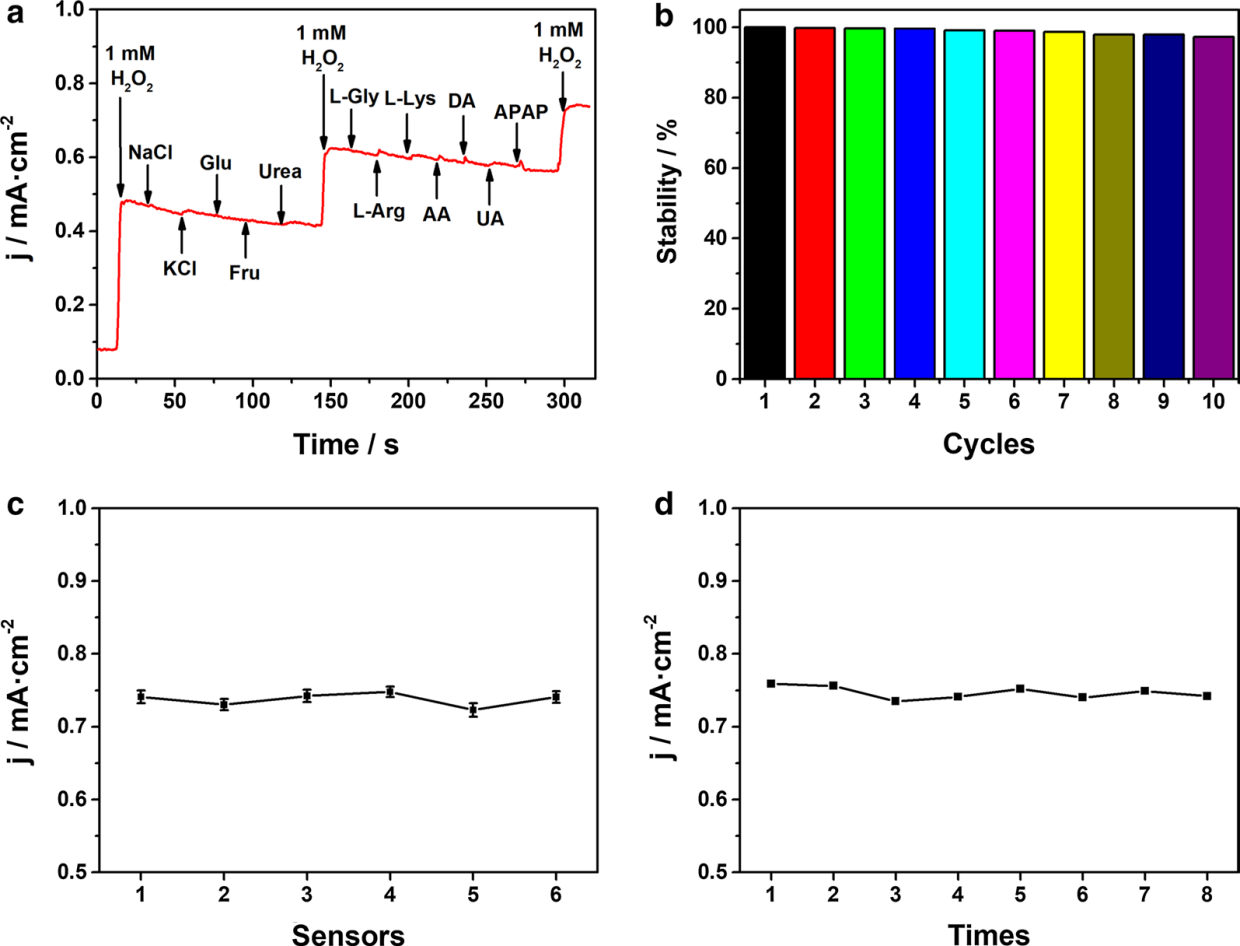
**a** Amperometric responses of Co_2_P/ITO electrode with the addition of 1 mM H_2_O_2_ and other interfering species (10 mM NaCl, KCl, Glu, Fru, urea, L-Gly, L-Arg, L-Lys, AA; 1 mM DA, UA; 0.5 mM APAP) in 0.1 M PBS. **b** The cathodic peak currents of ten successive scanning CV curves in 50 μM H_2_O_2_. **c** Reproducibility of six Co_2_P/ITO electrodes for detecting 1.0 mM H_2_O_2_. **d** Repeatability of Co_2_P/ITO electrode for detecting 1.0 mM H_2_O_2_ eight times

Anti-interference performance is another important property of biosensor. High purity nitrogen was utilized to avoid the influence by dissolved oxygen in solution because oxygen could be reduced at similar potential which was applied in amperometric test [51]. Comparing the CV curves of Co_2_P/ITO in 0.1 M PBS with or without nitrogen purging, the reduction potential and the current response of 2.5 mM H_2_O_2_ are similar, as shown in Fig. S8, which therefore suggests that the interference of dissolved oxygen can be neglected. Selectivity of Co_2_P/ITO was also tested with common substances and other small molecules in body fluid, such as some inorganic salts, saccharides, amino acids and reductive biomolecules. As shown in Fig. 4a, the current response after adding the above interferents can be neglected compared with the response of 1.0 mM H_2_O_2_. As both two O atoms of H_2_O_2_ could be bonded with one or two Co atoms [52], the H_2_O_2_ molecule would chemically adsorb on Co(II) species in Co_2_P specifically. In addition, the interference from indiscriminate oxidation of some reductive compounds in real biological samples at high potential can be also reduced significantly at lower bias potential [53]. Therefore, the favorable selectivity of Co_2_P toward H_2_O_2_ mainly benefits from the Co(II) species as specific adsorption sites and the applied negative bias potential during sensing process.

Moreover, the stability, reproducibility and repeatability of the Co_2_P/ITO transducer were also evaluated. The reduction peak currents of ten successive scanning CV curves in 50 μM H_2_O_2_ is shown in Fig. 4b. After ten cycles, the peak current of the electrode only fell by 2.7%. In addition, the sensor remained about 98.2% of its initial current response after being stored in air for one month (Fig. S9), demonstrating ideal detecting stability and outstanding long-term durability. The electrode-to-electrode reproducibility is investigated by calculating the relative standard deviation (RSD) of H_2_O_2_ current responses. To eliminate the potential error from electrode fabrication as far as possible, the steady current density in the presence of H_2_O_2_ is subtracted by the initial background signal of individual electrode and the obtained difference value is regarded as the electrochemical response of each electrode. Six Co_2_P/ITO electrodes were fabricated under the same conditions for controlled experiments and the RSD of current responses was 1.24%, as shown in Fig. 4c, indicating the relatively excellent reproducibility of Co_2_P/ITO. Meanwhile, repeatability was measured in one electrode by detecting 1.0 mM H_2_O_2_ eight times, and the RSD of 1.14% was achieved (Fig. 4d). The above results illustrate the satisfactory stability, reproducibility and repeatability of the electrode for non-enzymatic electrochemical detection of H_2_O_2_.

## Conclusion

In summary, Co_2_P NPs were successfully synthesized by hydrothermal method. Furthermore, the Co_2_P NPs prepared at 200 °C for 12 h have been proved as an efficient catalyst toward electrochemical reduction of H_2_O_2_ in pH 7.4 PBS. As a non-enzymatic H_2_O_2_ sensor, the Co_2_P/ITO electrode displayed a rapid amperometric response less than 5 s, a broader response range from 0.001 to 10.0 mM and a low detection limit of 0.65 μM, as well as satisfactory selectivity, reproducibility and stability. This work aims to broaden the research about the application of transition metal phosphide in electrochemical detection of small biomolecules and our Co_2_P/ITO sensor could be designed as a new non-enzymatic platform for H_2_O_2_ detection.

## Supplementary information

**Additional file 1**. **Fig. S1.** XRD patterns of Co_2_P NPs synthesized with different reaction times at 200 °C. **Fig. S2.** XPS survey spectrum of Co_2_P. **Fig. S3.** EDX spectra of Co_2_P NPs. **Fig. S4.** Amperometric responses of Co_2_P/ITO electrodes prepared at (a) different temperatures and (c) different times with successive addition of H_2_O_2_ in 0.1 M PBS. (b), (d) The calibration curve of steady current versus the concentration of H_2_O_2_. **Fig. S5.** The linear relationship between current density and concentration of H_2_O_2_ in different concentration ranges (a) 0.0001–1.0 mM, (b) 1.0–5.0 mM, (c) 5.0–10.0 mM. **Fig. S6.** Comparison of electrochemical properties between Co_2_P and Co(PO_3_)_2_. (a) LSV curves of Co_2_P and Co(PO_3_)_2_ modified electrode in 0.1 M PBS with and without 2.5 mM H_2_O_2_ at a scan rate of 100 mV s^−1^. (b) Nyquist plots of bare ITO, Co_2_P/ITO and Co(PO_3_)_2_/ITO electrode (electrolyte: 5.0 mM K_3_[Fe(CN)_6_]/ K_4_[Fe(CN)_6_] and 0.1 M KCl; bias: open circuit potential, amplitude: 5 mV, frequency range: 100 kHz ~ 0.01 Hz). **Fig. S7.** The linear relationship between current density and concentration of H_2_O_2_ in the physiological range. **Fig. S8.** CVs for Co_2_P/ITO electrode in 0.1 M PBS with or without N_2_ purging at a scan rate of 100 mV s^−1^. **Fig. S9.** CV responses at a scan rate of 100 mV s^−1^ in 0.1 M PBS containing 0.1 mM H_2_O_2_ of a Co_2_P/ITO electrode before and after being stored in air for one month. **Table S1**. The comparison on H_2_O_2_ sensing performance of the bare ITO electrode and the prepared Co_2_P sample at various reaction temperature.

## Data Availability

All data and materials are fully available without restriction.

## Abbreviations

NPs: Nanoparticles
ITO: Indium tin oxide
TEM: Transmission electron microscopy
HRTEM: High-resolution transmission electron microscopy
XRD: X-ray diffraction
XPS: X-ray photoelectron spectroscopy
EDX: Energy-dispersive X-ray spectroscopy
CV: Cyclic voltammetry
I-t: Amperometry
Gly: Glycine
AA: Ascorbic acid
UA: Uric acid
Arg: Arginine
Lys: Lysine
DA: Dopamine
APAP: Acetaminophen
ATMP: Trimethylene phosphonic acid
PBS: Phosphate buffer
LOD: Detection limit
RSD: Relative standard deviation
