# Supplementary material for: Cobalt Phosphide (Co2P) with Notable Electrocatalytic Activity Designed for Sensitive and Selective Enzymeless Bioanalysis of Hydrogen Peroxide
Source: Nanoscale Res Lett. 2021 Jan 13;16:11. doi: 10.1186/s11671-020-03469-9 (PMC7803862; doi:10.1186/s11671-020-03469-9)
Supplement: Supplementary file 1 — Additional file 1. Fig. S1. XRD patterns of Co2P NPs synthesized with different reaction times at 200 °C. Fig. S2. XPS survey spectrum of Co2P. Fig. S3. EDX spectra of Co2P NPs. Fig. S4. Amperometric responses of Co2P/ITO electrodes prepared at (a) different temperatures and (c) different times with successive addition of H2O2 in 0.1 M PBS. (b), (d) The calibration curve of steady current versus the concentration of H2O2. Fig. S5. The linear relationship between current density and concentration of H2O2 in different concentration ranges (a) 0.0001–1.0 mM, (b) 1.0–5.0 mM, (c) 5.0–10.0 mM. Fig. S6. Comparison of electrochemical properties between Co2P and Co(PO3)2. (a) LSV curves of Co2P and Co(PO3)2 modified electrode in 0.1 M PBS with and without 2.5 mM H2O2 at a scan rate of 100 mV s−1. (b) Nyquist plots of bare ITO, Co2P/ITO and Co(PO3)2/ITO electrode (electrolyte: 5.0 mM K3[Fe(CN)6]/ K4[Fe(CN)6] and 0.1 M KCl; bias: open circuit potential, amplitude: 5 mV, frequency range: 100 kHz ~ 0.01 Hz). Fig. S7. The linear relationship between current density and concentration of H2O2 in the physiological range. Fig. S8. CVs for Co2P/ITO electrode in 0.1 M PBS with or without N2 purging at a scan rate of 100 mV s−1. Fig. S9. CV responses at a scan rate of 100 mV s−1 in 0.1 M PBS containing 0.1 mM H2O2 of a Co2P/ITO electrode before and after being stored in air for one month. Table S1. The comparison on H2O2 sensing performance of the bare ITO electrode and the prepared Co2P sample at various reaction temperature. [file 11671_2020_3469_MOESM1_ESM.docx]

**Supporting information**

**Cobalt phosphide (Co_2_P) with notable electrocatalytic activity designed for sensitive and selective enzymeless bioanalysis of hydrogen peroxide**

Donghang Yin^1^, Junyan Tang^1^, Rongbiao Bai^1^, Shuyi Yin^1^, Mengnan Jiang^1^, Zigui Kan^1^, Hongmei Li^1^, Fei Wang^1*^ and Caolong Li^1,2*^

*^1^ Key Laboratory of Biomedical Functional Materials, School of Science, China Pharmaceutical University, Nanjing, 211198, P. R. China.*

*^2^ Tibetan Medicine Research Institute, Tibetan Traditional Medical College, Tibet, 850000, P. R. China*


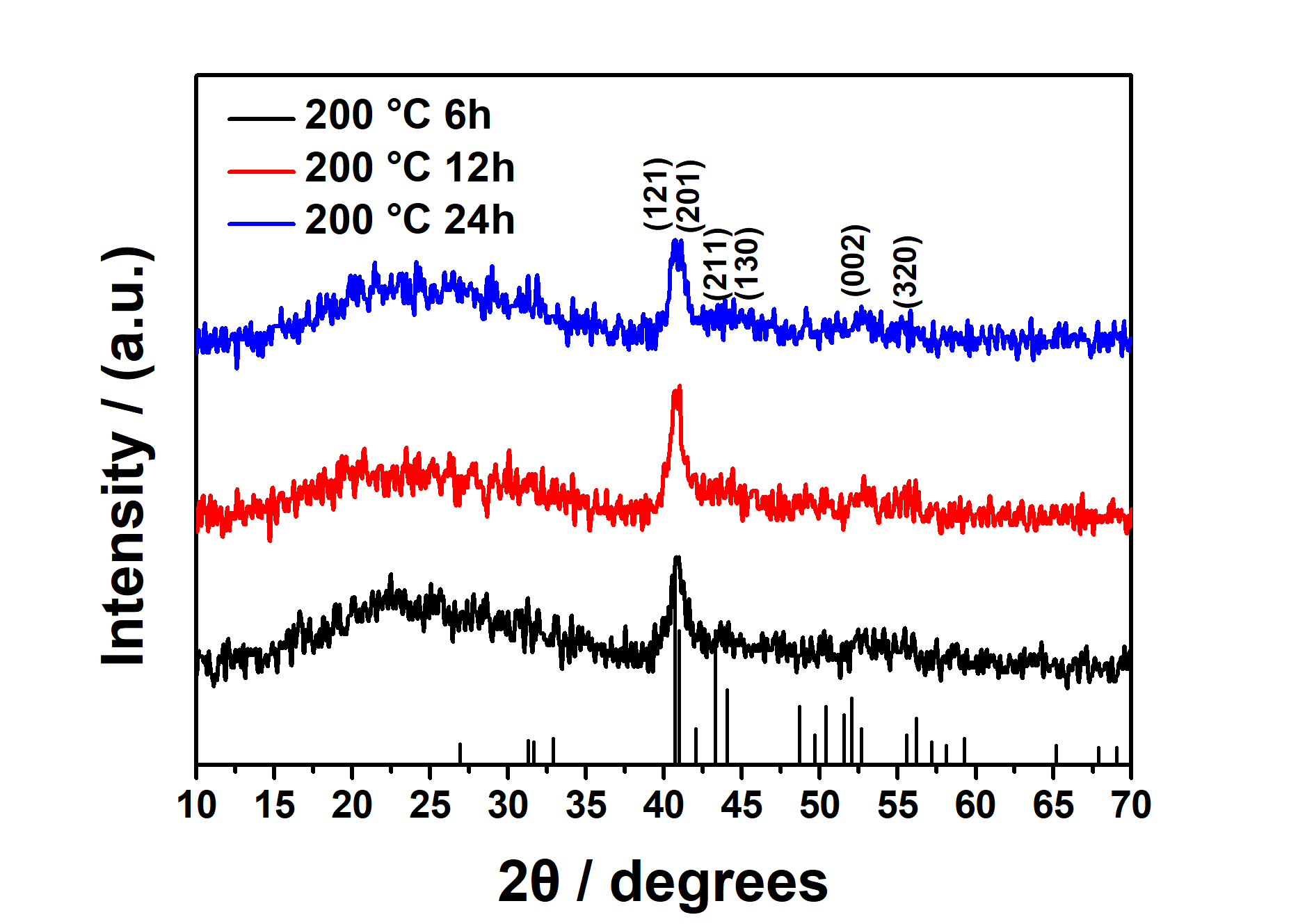


**Fig. S1.** XRD patterns of Co_2_P NPs synthesized with different reaction times at 200 °C.


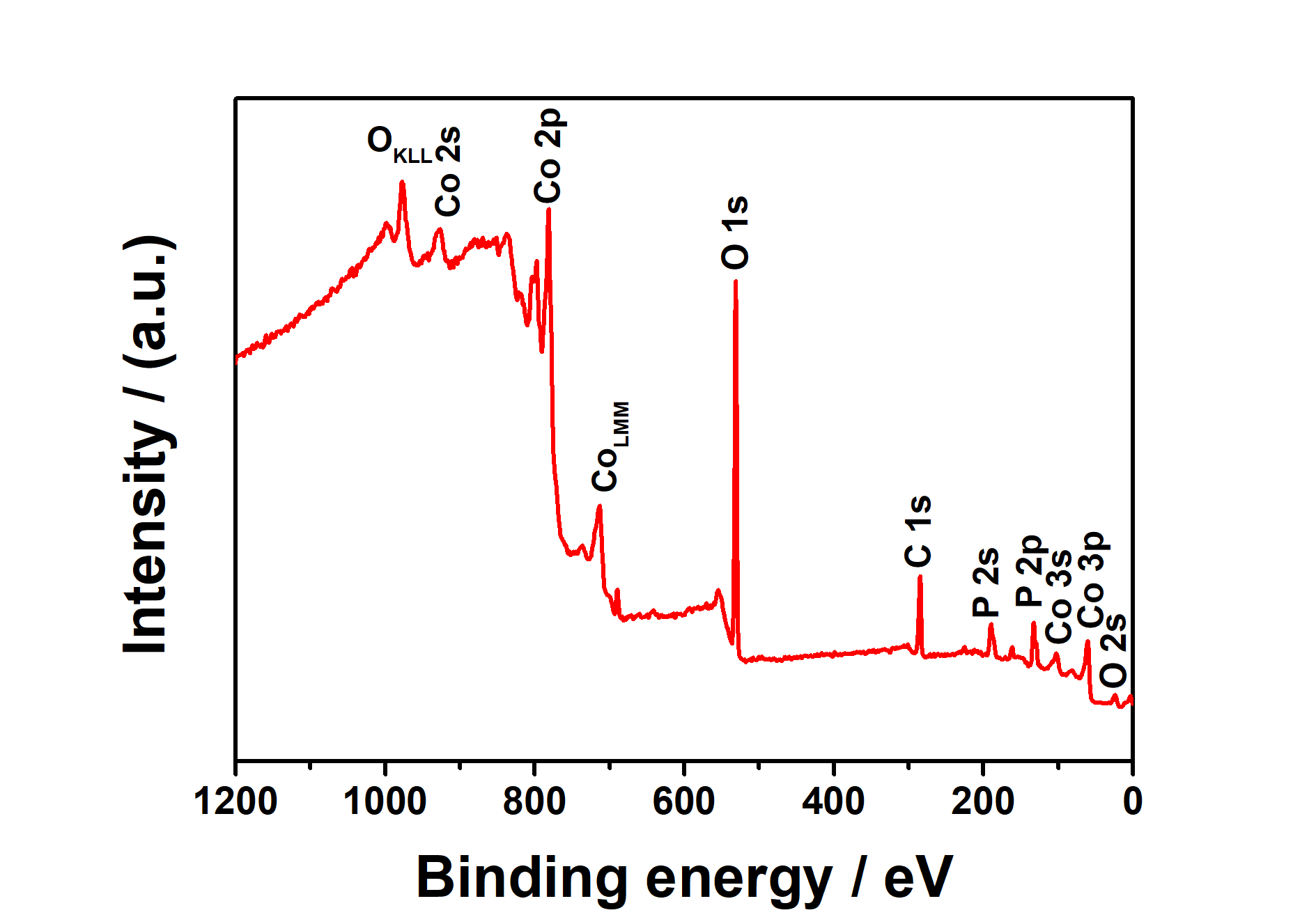


**Fig. S2.** XPS survey spectrum of Co_2_P.


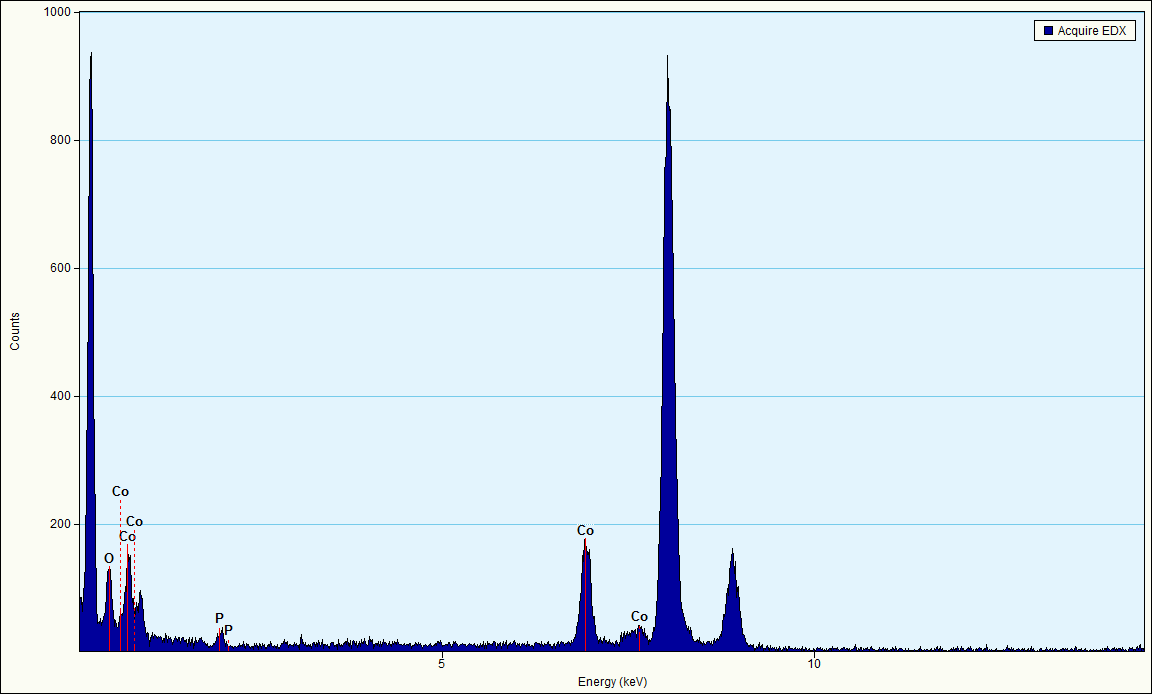


**Fig. S3.** Energy dispersive X-ray spectroscopy (EDX) spectra of Co_2_P NPs.


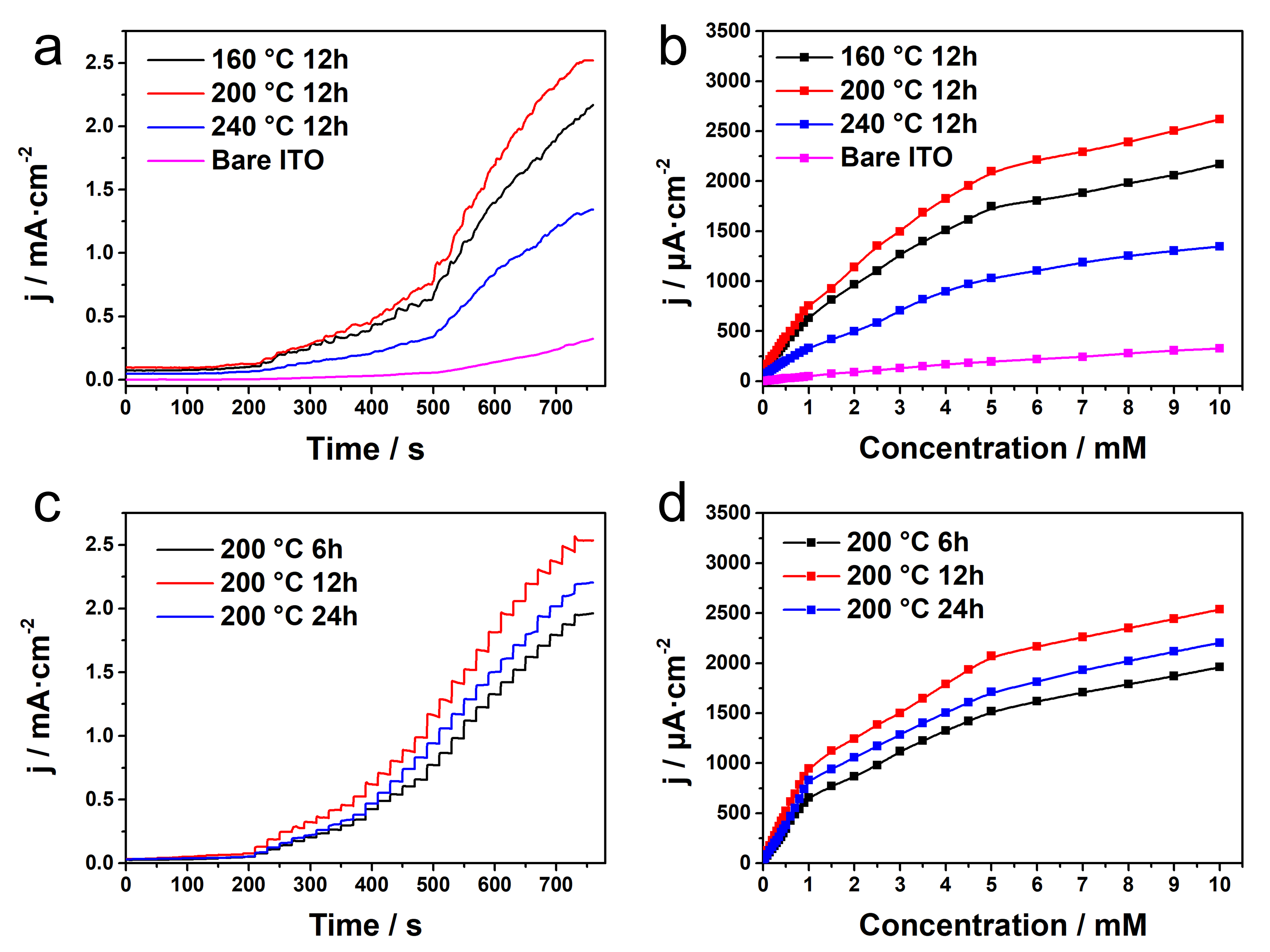


**Fig. S4.** Amperometric responses of Co_2_P/ITO electrodes prepared at (a) different temperatures and (c) different times with successive addition of H_2_O_2_ in 0.1 M PBS. (b), (d) The calibration curve of steady current versus the concentration of H_2_O_2_.


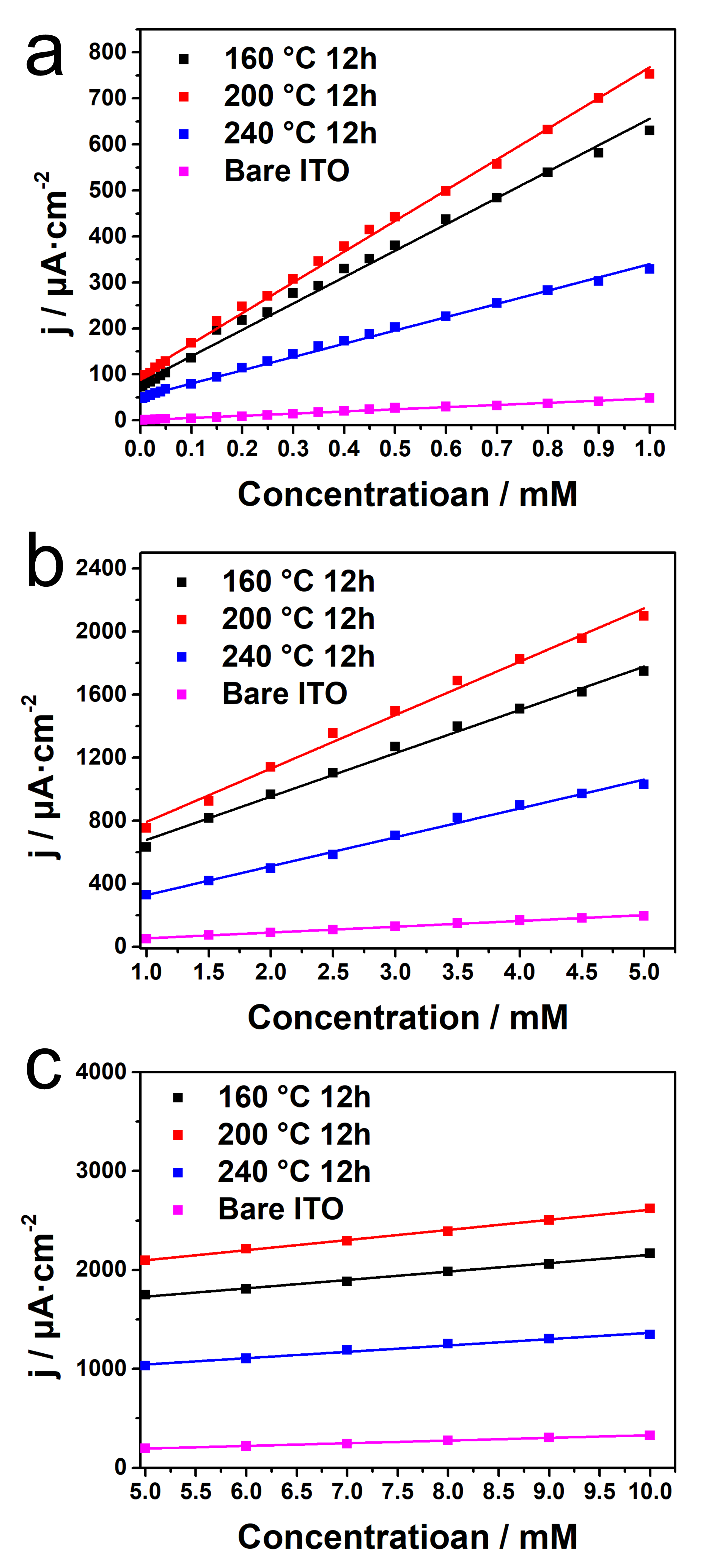


**Fig. S5.** The linear relationship between current density and concentration of H_2_O_2_ in different concentration ranges (a) 0.0001-1.0 mM, (b) 1.0-5.0 mM, (c) 5.0-10.0 mM.

**Table S1**. The comparison on H_2_O_2_ sensing performance of the bare ITO electrode and the prepared Co_2_P sample at various reaction temperature.

| Electrode | Linear range (mM) | Sensitivity (*μ*A mM^-1^ cm^-2^) | R^2^ |
| --- | --- | --- | --- |
| 160 °C, 12 h Co_2_P/ITO | 0.0001-1.0 | 573.9 | 0.9940 |
|  | 1.0-5.0 | 275.0 | 0.9932 |
|  | 5.0-10.0 | 84.7 | 0.9893 |
| 200 °C, 12 h Co_2_P/ITO | 0.0001-1.0 | 668.6 | 0.9982 |
|  | 1.0-5.0 | 339.0 | 0.9920 |
|  | 5.0-10.0 | 102.3 | 0.9953 |
| 240 °C, 12h Co_2_P/ITO | 0.0001-1.0 | 228.7 | 0.9966 |
|  | 1.0-5.0 | 183.3 | 0.9929 |
|  | 5.0-10.0 | 64.2 | 0.9813 |
| Bare ITO | 0.0001-1.0 | 47.0 | 0.9939 |
|  | 1.0-5.0 | 36.8 | 0.9979 |
|  | 5.0-10.0 | 27.1 | 0.9943 |

When the hydrothermal synthetic time was controlled within 12 h, the Co_2_P/ITO prepared at 200 °C showed the highest current response to H_2_O_2_ reduction compared to those from other temperature and bare ITO (Fig. S4a, S4b). The comparison of detection sensitivity is shown in Fig. S5 and Table S1. It should be noticed that the sensitivity of Co_2_P/ITO prepared at 200 °C is higher than that of the samples prepared at other temperatures and bare ITO. Combining with XRD results, the higher purity and better crystallinity of Co_2_P prepared at 200 °C may contribute to the best electrocatalytic activity in H_2_O_2_ reduction. Fig. S4c and S4d show the amperometric responses of Co_2_P/ITO prepared at 200 °C for 6, 12 and 24 h, respectively. The current density and sensitivity of the 12 h-sample is highest among the other samples. The XRD patterns of Co_2_P in Fig. S1 also inferred that better crystalline may benefit the improvement of electrocatalytic activity. Therefore, we choose the Co_2_P sample prepared at 200 °C for 12 h as the best sensing material for the electrochemical detection of H_2_O_2_.


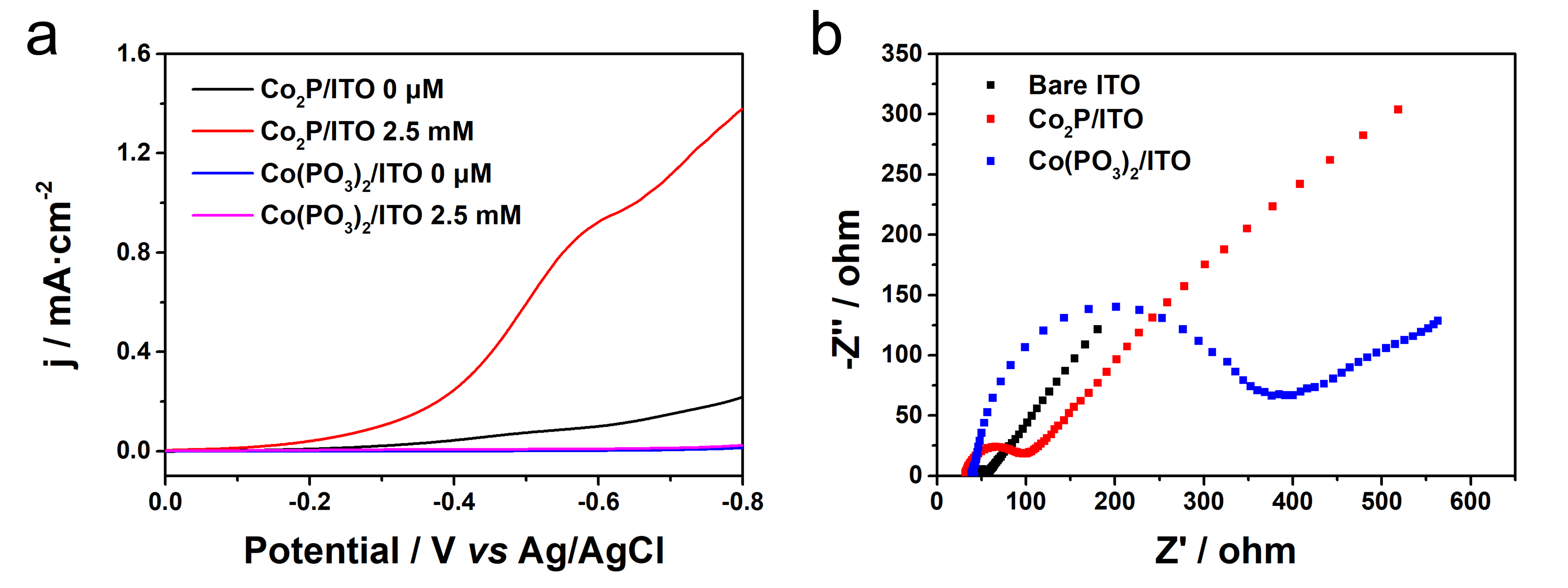


**Fig. S6.** Comparison of electrochemical properties between Co_2_P and Co(PO_3_)_2_. (a) Linear sweep voltammetry (LSV) curves of Co_2_P and Co(PO_3_)_2_ modified electrode in 0.1 M PBS with and without 2.5 mM H_2_O_2_ at a scan rate of 100 mV s^-1^. (b) Nyquist plots of bare ITO, Co_2_P/ITO and Co(PO_3_)_2_/ITO electrode (electrolyte: 5.0 mM K_3_[Fe(CN)_6_]/ K_4_[Fe(CN)_6_] and 0.1 M KCl; bias: open circuit potential, amplitude: 5 mV, frequency range: 100 kHz ~ 0.01 Hz).


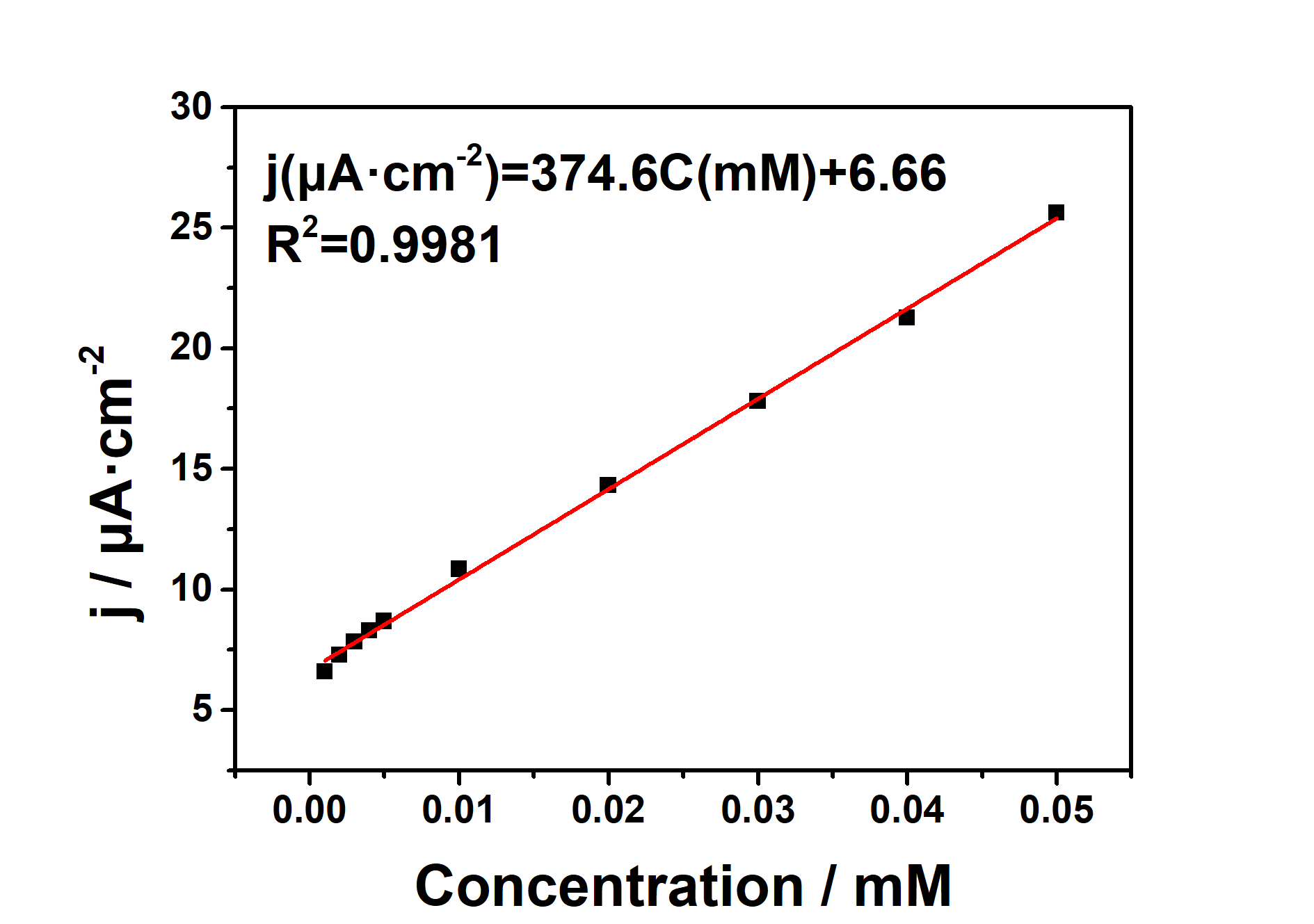


**Fig. S7.** The linear relationship between current density and concentration of H_2_O_2_ in the physiological range.


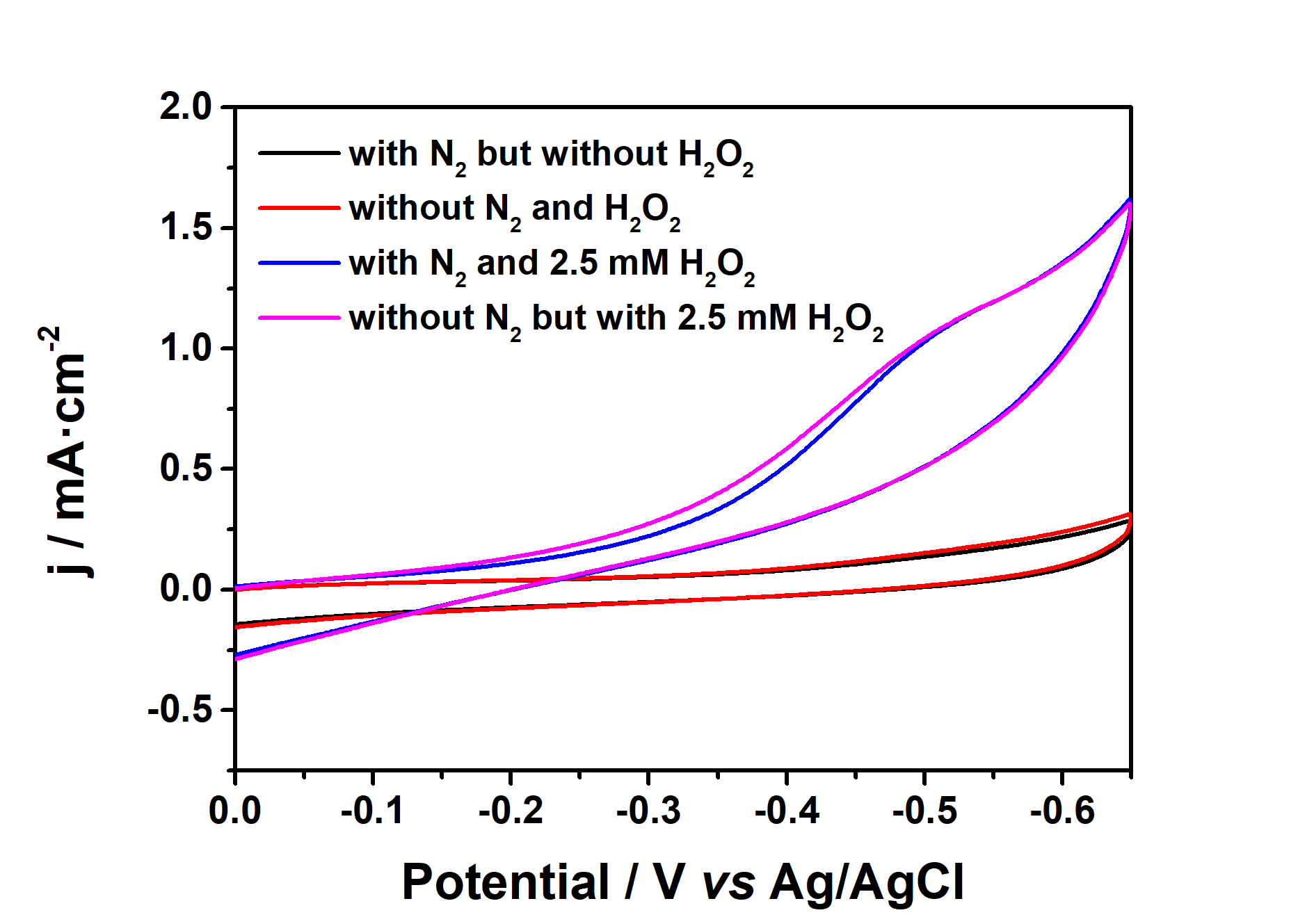


**Fig. S8.** CVs for Co_2_P/ITO electrode in 0.1 M PBS with or without N_2_ purging at a scan rate of 100 mV s^-1^.


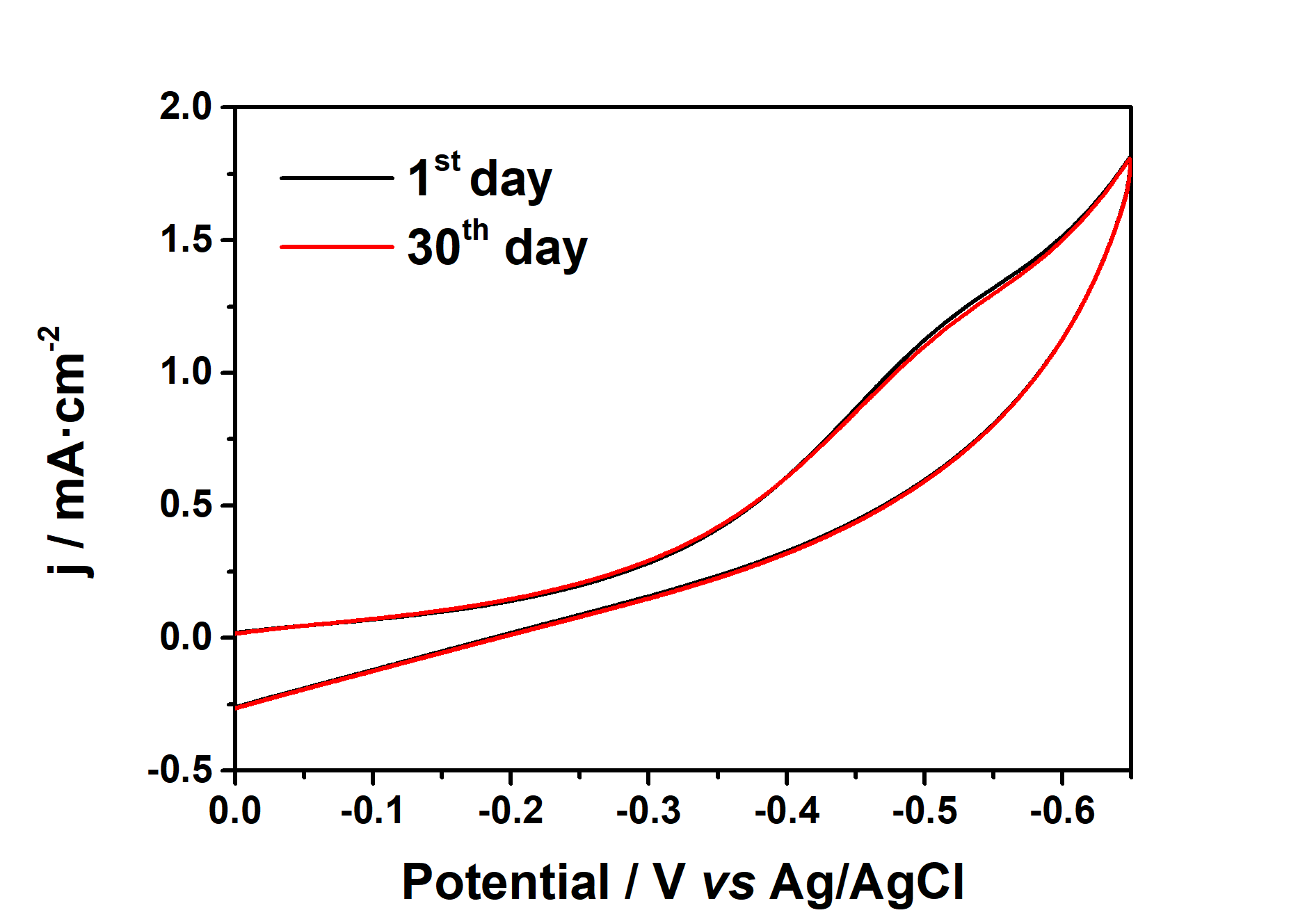


**Fig. S9.** CV responses at a scan rate of 100 mV s^-1^ in 0.1 M PBS containing 2.5 mM H_2_O_2_ of a Co_2_P/ITO electrode before and after being stored in air for one month.
